# Exploring the Feasibility of Combining Chronic Disease Patient Registry Data to Monitor the Status of Diabetes Care

**Published:** 2008-09-15

**Authors:** Angela M Kemple, Noelle Hartwick, Marilyn H Sitaker, Jeanne J Harmon, Jan Norman, Kathleen Clark

**Affiliations:** Chronic Disease Prevention Unit, Washington State Department of Health; Chronic Disease Prevention Unit, Diabetes Prevention and Control Program, Washington State Department of Health, Olympia, Washington; Chronic Disease Prevention Unit, Diabetes Prevention and Control Program, Washington State Department of Health, Olympia, Washington; Chronic Disease Prevention Unit, Diabetes Prevention and Control Program, Washington State Department of Health, Olympia, Washington; Chronic Disease Prevention Unit, Diabetes Prevention and Control Program, Washington State Department of Health, Olympia, Washington; Health Care Authority, Olympia, Washington. At the time of this project, Ms Clark was manager of the Washington State Diabetes Prevention and Control Program

## Abstract

**Introduction:**

To provide direction and to support improvements in diabetes care, states must be able to measure the effectiveness of interventions and gain feedback on progress. We wanted to know if data from multiple health clinics that are implementing quality improvement strategies could be combined to provide useful measurements of diabetes care processes and control of intermediate outcomes.

**Methods:**

We combined and analyzed electronic patient health data from clinic sites across Washington State that used the Chronic Disease Electronic Management System (CDEMS) registry. The data were used to determine whether national and state objectives for diabetes care were met. We calculated the percentage of patients that met standards of care in 2004.

**Results:**

The pooled dataset included 17,349 adult patients with diabetes from 90 clinics. More than half of patients were above recommended target levels for hemoglobin A1c testing, foot examination, hemoglobin A1c control, and low-density lipoprotein cholesterol control. Fewer patients met recommendations for nephropathy assessment, eye examinations, and blood pressure control. In terms of meeting these standards, rates of diabetes care varied across clinics. CDEMS rates of care were compared with those reported by other data sources, but no consistent pattern of similarities or differences emerged.

**Conclusion:**

With committed staff time, provider support, and resources, data from clinical information systems like CDEMS can be combined to address a deficiency in state-level diabetes surveillance and evaluation systems — specifically, the inability to capture clinical biometric values to measure intermediate health outcomes. These data can complement other surveillance and evaluation data sources to help provide a better picture of diabetes care in a state.

## Introduction

Diabetes is a growing public health problem (1), but care continues to be less than optimal (2-4), despite evidence that effective and economical interventions can result in fewer complications and improved outcomes (5-7). Therefore, the Institute of Medicine labeled diabetes as a priority area for quality improvement in the United States (8) and suggested substantial changes in and redesign of health care systems (9), including the better use of information technology to monitor health care.

National and state objectives for diabetes care are used to evaluate the effectiveness of prevention and intervention activities (10). Local targets for these objectives have been set by each state's Diabetes Prevention and Control Program (DPCP). Objectives for the Washington State DPCP focus on increasing population-level rates of process measures (annual foot and eye examinations, biannual hemoglobin A1c [HbA1c] tests, annual nephropathy assessment, annual influenza vaccination, and previous pneumococcal vaccination) and intermediate outcomes (controlled levels of HbA1c, blood pressure, and low-density lipoprotein [LDL] cholesterol).

States must be able to measure the cumulative effect of broad community, health system, and health communication interventions and to monitor progress toward diabetes objectives over time. Aggregate data from individual clinical information systems have been used by large organizations, including health care systems, federal health care organizations, and community health centers to monitor, coordinate, and manage care for targeted diabetes populations (11-22). State health departments, however, have used these kinds of data to only a limited degree (23). The lack of state-specific surveillance data for measuring progress on 3 diabetes indicators — glucose, lipid, and blood pressure control — is a deficiency in evidence supporting the impact of a state health department's effort to improve diabetes outcomes.

In 2005, the Washington State DPCP assessed its progress toward meeting state and national diabetes objectives to determine whether established targets for each objective needed to be modified. An extensive review of population-based data was done to ascertain what data sources could be incorporated into the surveillance program to help track progress toward meeting objectives. Whereas the DPCP regularly uses statewide BRFSS (Behavioral Risk Factor Surveillance System) telephone survey data to monitor processes of care (foot examination, eye examination, HbA1c testing, and influenza and pneumococcal vaccinations), no statewide source exists for collecting information on nephropathy screening and intermediate health outcomes (glucose, lipid, and blood pressure control). For this reason, the DPCP decided to explore the feasibility of obtaining data from a patient registry known as the Chronic Disease Electronic Management System (CDEMS), which is used by primary care providers across Washington (24). CDEMS is the only source of state-specific data available to the DPCP to monitor nephropathy screening and HbA1c, LDL, and blood pressure values among a large patient population with diabetes. Consolidated data from all Washington clinics using CDEMS covered approximately 13% of all state residents with diabetes in 2004.

Our goals for this data consolidation project were to 1) measure the status of patients in CDEMS registries in terms of meeting state and national objectives for diabetes, 2) provide aggregate comparison data for individual clinics using CDEMS, and 3) determine the feasibility of combining and using clinic data for ongoing diabetes surveillance and evaluation efforts in Washington. This report focuses on the process of aggregating registry data, resources used, initial outcomes, lessons learned, and the utility of combining clinic data for future endeavors.

## Methods

DPCP program staff, Washington State Department of Health (DOH) project epidemiologists, and 2 contractors worked together to plan, coordinate collection of, consolidate, and cleanse CDEMS data. The DPCP contracted with the CDEMS technical support consultant and with Krupski Consulting, Inc, Olympia, Washington, a firm that extracts, transforms, and loads data (25). The Appendix provides details on the tasks, estimated time, and cost for this project.

At the start of this project, epidemiologists spent considerable time working with DPCP staff and the CDEMS technical consultant to gain a thorough understanding of the development philosophy, implementation, maintenance, and structure of CDEMS and variations between clinics to guide clinic recruitment and subsequent data consolidation. CDEMS is a Microsoft Access database application developed by the Washington State DPCP in 2002 (24). It is available at no cost to all who wish to use it (http://www.cdems.com). The program was designed to help medical providers, clinic managers, and other health care staff track the care of patients with chronic health conditions. CDEMS stores individual patient demographic information, visit dates, vital signs, medications, diagnoses, services, laboratory results, and custom notes in 7 main data tables. Data entry screens, such as the Patient Information Record and New Visit Form (Figure), are used to populate the main tables in the database. The CDEMS registry has predefined data codes to track diabetes care, but these can be modified and measures added to monitor other chronic conditions. A laboratory interface is available to download results from several major laboratories electronically. Printed progress notes, patient lists, and summary reports are generated from the registry database to help deliver services more efficiently and effectively and to monitor changes from quality improvement efforts.

**Figure.**
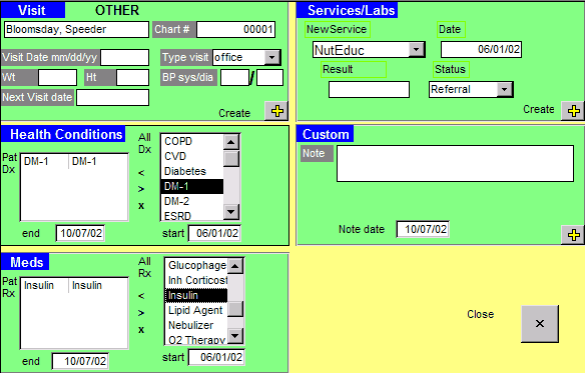
New Visit Form With Example Patient Data, Chronic Disease Electronic Management System, Washington State Department of Health, 2002. Published with permission.

CDEMS registries are used predominately in primary care clinics in community and rural settings and in Indian Health Service clinics throughout the state. Most clinics began using CDEMS as part of the Washington State Collaborative (14,26) or National Health Disparities Collaborative (27). These collaboratives use a proactive approach that offers proven strategies to help primary care practice teams manage care for people with chronic diseases. For this reason, we considered most patients in CDEMS to be potentially better managed than the general diabetes population in Washington state.

Clinics populated their registries by either entering data on new patients prospectively, entering data from review of medical records retrospectively, or importing data from other systems. Some clinics populated their registries with a subset of their patients, whereas others used their total diabetes patient population. Demographic data were often imported from a billing system and were complemented by data abstracted from medical records, to capture recent and historical health information going back 1 to 2 years. At the time of this project, only 22% of clinics used laboratory interfaces to download laboratory results electronically.

Because of variations in how clinics used data fields in CDEMS, we compiled a master list of data fields associated with each table in the registry, which included only those that would be useful for measuring progress toward state and national objectives.

Because CDEMS stores individually identifiable health information, protection under the Health Insurance Portability and Accountability Act (HIPAA) was reviewed by the HIPAA officer for the Washington State DOH. The project was designated as public health surveillance and considered to be in compliance with HIPAA privacy rule requirements (28). The Washington State DOH Information Technology Security Office conducted an assessment of data confidentiality and sensitivity before clinic recruitment and data aggregation.

To minimize the data transfer burden and encourage response, we asked clinics to simply copy their registry database, including all patient records, data fields, and years available, onto a CD-ROM, DVD, or floppy disk and send it directly to the DPCP in a return postage-paid mailing envelope. We recruited clinics by e-mail initially, followed up with a formal letter explaining the project, and contacted nonresponding clinics by e-mail and telephone. Clinics that participated in the project were later provided a summary of results from the combined CDEMS database to compare with their own data.

### Collecting, combining, and cleansing data

The CDEMS technical support contractor coordinated the collection and transfer of databases from the clinics to the DPCP. DPCP staff logged clinics' CDEMS databases by date of arrival before forwarding data to Krupski Consulting for consolidation. This contractor worked closely with project staff and the epidemiologists to identify the relevant CDEMS variables and data fields needed to assess the number of patients meeting state and national objectives. Clinics with multiple locations submitted data inconsistently, some providing a database for each location and others combining locations into 1 large database. In addition, some clinics included only a subset of their total patient population. As data were received, project staff created a unique code to identify the source of data.

The aim of data consolidation was to combine the records from source tables in each clinic's registry into a single master database containing values with consistent meaning. Because CDEMS was designed to be adaptable to each clinic, clinics entered data in various ways, and thus transforming and cleansing the data before aggregation was time-consuming. Values for each field in the CDEMS database were standardized to reconcile variations between clinics. For example, *glucose* in the original datasets received from each clinic may have been recorded as *Glu*, *GLU*, *glu*, *glucose*, or *Glucose*; all terms were changed to *glucose* in the aggregate database. In addition, the contractor ensured that codes predefined in the original databases for diagnosis, service, and laboratory fields were incorporated into the aggregate database and dealt with formatting issues.

To make subsequent data analysis more efficient, the contractor worked with project staff and the epidemiologists to combine and recode similar values. For example, all insurance plan names in the database were recoded to 5 unique values — commercial/private, Medicaid, Medicare, self-pay, or no insurance. Some field values were automatically assigned, but others had to be done manually, case-by-case. Unknown or invalid values were set to null in the condensed database. To determine health conditions alone, we examined and recoded more than 130 different data field values. More than 200 different field values were reviewed and recoded for health services, more than 500 values for laboratory results, and more than 2,000 values for health care coverage. The contractor added new fields to various tables in the combined database to document how original values were recoded.

The contractor applied proprietary software tools to consolidate data efficiently and cost-effectively. Afterward, the epidemiologists reviewed data, removed duplicate databases, and organized and linked data tables for analysis. Following these activities, 493 records (<1%) were excluded from further analysis.

Diabetes was recorded in the CDEMS table that stores information on a patient's health conditions by clinic staff if a patient had an *International Classification of Diseases, 9th Revision, Clinical Modification* (ICD-9-CM) diagnosis code of diabetes type 1 or type 2 (confirmed through chart audit) and a date of diagnosis. For example, if a patient had diabetes, a provider would select a preset health condition code for diabetes (Diabetes, DM-1, or DM-2) under the health conditions section in the CDEMS New Visit Form (Figure). For our analysis, we selected adult patients with diabetes who met the following criteria: 1) had at least 1 visit, service, or laboratory result in 2004, 2) were diagnosed with diabetes before 2004 so they had a full year to receive services, and 3) were at least 18 years old. Patients with gestational diabetes or prediabetes were excluded. The final pooled CDEMS database included 51,233 patients, of which 17,349 met these criteria.

### Measures

We were able to adequately assess the indicators described in Table 1, which lists the indicator definitions and reporting ranges we selected before analysis. We were unable to assess receipt of annual influenza vaccine and previous pneumococcal vaccination because patients are usually referred to other facilities for vaccinations, and few clinics have a feedback system to monitor outside services. Further, most clinics did not collect historical information on lifetime pneumococcal vaccination.

### Statistical analysis

The data were analyzed using Microsoft Access 2003 (Microsoft Corp, Redmond, Washington) and Stata statistical software, version 9.0 (StataCorp LP, College Station, Texas). Percentages and 95% confidence intervals were calculated using the binomial Wald method. Median percentages and ranges across clinics were also calculated because of substantial variation in rates of meeting diabetes care objectives among clinics.

## Results

Most of the 132 eligible Washington clinics (85%) submitted data for this project. More than two-thirds (68%) provided data that were included in final sample of 17,349 adult patients with diabetes from 90 primary care office settings. Clinics in the combined database ranged in size from 1 to 2,483 patients with diabetes in 2004. More than 90% of clinics participated in a collaborative or were a satellite clinic of an organization that participated in a collaborative. Approximately 40% were community health centers or federally qualified community health centers.

We excluded data from 22 clinics (17%) from our analysis because the clinic database could not be opened or combined, the clinic's registry was not implemented at the start of the project period, or the clinic's data collection and reporting methods made it difficult to identify diabetes patients. No particular pattern was noted with the information available from these clinics compared with that from clinics included in the combined database.

Twenty clinics (15%) did not participate in the project because they did not start their registry until late in 2004, had staff turnover involving the CDEMS coordinator at the clinic, or lacked time. We had insufficient information to compare patient populations between CDEMS clinics that submitted data and CDEMS clinics that did not. The clinics that did not participate came mostly from nonurban areas, but we observed no further differences such as private vs public status, participation in the Washington State Collaborative, or geographic location.

The average age of patients with diabetes in the combined database was 59 years (range 18-100 years). Slightly more than half (53%) were female. Race/ethnicity was documented for only 60% of patients, 59% of whom were listed as white, 21% Hispanic, 8% African American, 6% Asian, 3% American Indian/Alaska Native, 2% Native Hawaiian/Other Pacific Islander, and less than 1% other race. Approximately 38% of the participants were commercially insured, 22% had Medicare, 19% had unknown insurance status, 8% had no health insurance, 9% had Medicaid, 5% were self-pay, and less than 1% had other sponsored care.

The age and sex distribution of adult patients with diabetes in the CDEMS database was different from that of the overall Washington adult diabetes population (Table 2). A larger proportion of CDEMS patients with diabetes were aged 65-74 years and a smaller proportion were aged 75 years or older compared with the overall population, and the proportion of women was greater among CDEMS patients. Hispanics and Asians appeared to be overrepresented in CDEMS compared with the state. However, because the race and ethnic origin of many patients was not recorded in CDEMS, we are unable to draw conclusions about the differences between these populations. Similarly, the large proportion of CDEMS patients listed as having "unknown insurance status" means that we are unable to comment on differences in health insurance coverage between CDEMS and the overall statewide populations.

Tables 3 and 4 show the distribution of processes of diabetes care and intermediate health outcomes among adult patients with diabetes in the aggregate database. More than 50% of patients were above recommended target levels for HbA1c testing, foot examination, HbA1c control, and LDL cholesterol control. Fewer patients met recommendations for nephropathy assessment, eye examinations, and blood pressure control. Performance on these indicators varied across clinics. Table 5 further describes the values for each of the intermediate health outcomes assessed.

Table 6 compares results from the consolidated CDEMS database with results from other state and national data sources. CDEMS patients had more favorable results for HbA1c and LDL cholesterol levels than did the overall population, and their results did not differ noticeably for receiving an HbA1c test, LDL cholesterol test, or nephropathy screen in the past year. CDEMS results were less favorable for annual foot examinations, annual eye examinations, and biannual HbA1c, compared with other data sources.

## Discussion

We sought to determine the feasibility of using aggregate clinic data for ongoing diabetes surveillance and evaluation efforts in Washington State. Our work shows that with committed staff time, provider support, and resources, data from clinical information systems like CDEMS can be combined to address a deficiency in state surveillance and evaluation systems — specifically, the inability to capture clinical values to measure intermediate health outcomes for diabetes. The intent is to use the CDEMS measures that do not appear in BRFSS to complement BRFSS data, with the understanding that one of the limits of the aggregate CDEMS database is that it reflects only 9% of the diabetes population — and they are probably specially managed because most clinics have received intensive training on implementing the Chronic Care Model (29,30).

CDEMS patients' better HbA1c and LDL cholesterol levels compared with the overall state population of people with diabetes (Table 6) may be because most CDEMS clinics were alumni of the Washington State Collaborative (14,26) or Health Disparities Collaborative (27) or were affiliated with a clinic that participated in a collaborative that focused on these measures.

CDEMS screening results for HbA1c, LDL cholesterol, or nephropathy in the past year were not noticeably different compared with data sources that were not restricted to specially managed populations. The CDEMS results also were not as good for annual foot examinations, annual eye examinations, and biannual HbA1c tests.

Some differences between the CDEMS results and other data sources could be attributable to differences in how measures are defined, how ranges for responses are defined, how data are collected (clinical data vs self-report), and how data are entered into systems, or they may represent a true difference in outcomes. For example, the low prevalence of receiving eye examinations in CDEMS compared with self-reported eye examination data from statewide BRFSS is not unexpected. Follow-up documentation on patients referred for eye examinations outside the care clinic is rare, and poor agreement between self-report and medical record data on annual eye examinations has been documented elsewhere (31). Without a detailed study comparing the data sources, clinics, patients who are captured within the data sources, or study methods, it is difficult to explain observed differences.

### Lessons learned from combining data

A project of this magnitude required a commitment from the state DPCP to ensure that financial and staff resources were available to complete the work. The project required substantial time, coordination, and communication from internal and external staff who assisted with project management and clinic recruitment; contractors and programmers, who managed data submission and consolidation; CDEMS staff, who provided technical support; and epidemiologists, who provided project coordination, project design, data consultation, and analysis.

Clinic recruitment was facilitated by established relationships between DPCP and CDEMS users through the ongoing technical assistance provided to clinics by DPCP as part of the Washington State Collaborative. We learned to work with the clinics' central registry coordinators (especially for multisite implementations) rather than each clinic within a larger system. It was also necessary to be explicit about which project we represented, since multiple quality improvement evaluation projects occurred simultaneously within the Washington State DOH.

We found we needed to provide several options for ensuring patient privacy and data security with the clinics and Washington State DOH information technology staff before arriving at a simple and acceptable process for gathering data. Although we initially favored a secure file transfer protocol Web site as a central repository for data submission, this option would have caused undue burden, compromising clinic participation. Even with the easier option of copying the databases to a CD, several clinics needed our assistance to transfer their data.

During data consolidation, we needed to complete several tasks to analyze data more efficiently (eg, reviewing various field codes from each clinic, combining similar values, handling different types of data, and identifying data fields to define measures). After data were combined, more time was spent identifying unique patients and removing duplicate data. Resolving duplications first may have minimized postconsolidation cleanup of the data and ensured accuracy of numerator and denominator counts required to estimate the percentage receiving care. It would have been useful to have a comprehensive codebook before analysis to identify field names and values, track programming used to combine data, and note changes made to the original data submitted.

After data were combined and reviewed, we still needed to modify some project measures. For example, because there is no standard method for reporting nephropathy results in CDEMS, we reviewed results manually to determine which met the definition for annual screening. The distinctive ability for the user to customize CDEMS led to variation in the data that required additional effort on our part to standardize before analysis.

### Limitations

Our project had several limitations. First, it may be biased toward better outcomes because participating clinics are engaged in quality improvement efforts, although quality improvement efforts may have focused on a few measures only, and specialty clinics are generally not represented in CDEMS. Second, in this initial look at the data, only unweighted aggregate population statistics are reported; thus, clinics with larger patient populations may disproportionately affect the results. Our combined data represent a convenience sample, and detailed information to construct sample weights was not available. In the future, additional time and resources will be required to collect detailed information on each clinic and account for differences between clinics. Third, individual clinic datasets reflect variations in entry protocols, reporting methods, field definitions, and years covered. Some clinics collected registry data for a few objectives during the project period. Our overall rates may have been higher had we been able to account for inconsistencies in data collection intensity and measurement standards across clinics. Because we were unable to identify new patients based on the start date field in CDEMS (ie, clinics used different definitions for "start date"), our results may include patients who did not participate throughout the entire project period. These limitations highlight the need for improved standards in CDEMS data collection and reporting.

### Implications

This project reflects the status of state and national objectives for approximately 9% of adults with diabetes in Washington in 2004. However, the number of patients tracked in CDEMS grew by 83% from 2004 to 2007 (32). Furthermore, provider use of CDEMS grew by 39% during the same period (32). As more providers use clinical information systems like CDEMS, the potential to gather more representative data will improve. This data quality will be necessary as provider accountability, pay-for-performance, and public reporting of quality measures are increasingly emphasized. How the growing use of electronic medical records (EMRs) will influence CDEMS use has yet to be determined, but some clinics in Washington use both CDEMS and EMR registries (17% in 2007) because of the limited usefulness of EMR systems to track patients with chronic conditions (32,33).

This project shows there is a need to improve standardization of CDEMS data entry and reporting for a minimum number of key measures to track progress over time, to provide appropriate and valid comparison data, and to help organizations to share knowledge about progress with one another. For example, establishing a consistent feedback loop and data controls to capture completed referrals for eye examinations would improve monitoring of this objective in the population.

### Conclusion

This project shows how one state combined individual clinic data from chronic disease registries as part of an overall effort to enhance its diabetes surveillance capacity. Being able to monitor the status of diabetes care, track changes, and conduct peer comparisons through the collection, combination, and use of clinic data may help stimulate health practitioners to implement broad systematic improvements and provide data to the DPCP for future program plans.

## Figures and Tables

**Table 1:** Indicator Definitions and Reporting Ranges Used to Analyze Consolidated Registry Data From the Washington State Chronic Disease Electronic Management System (CDEMS), 2004

Indicator (Definition^a^)	Valid Reporting Range(s)	Additional Edit Filters and Exclusion Criteria	Source
Annual foot examination (have at least 1 ICD-9-CM code for foot exam in 2004)	Not applicable	Only included patients with code indicating exam was completed. Examination with referral or declined status was excluded.	Used recommendations from the DPCP and CDEMS technical support staff.
Annual eye examination (have at least 1 ICD-9-CM code for eye exam in 2004)	Not applicable	Only included patients with code indicating exam was completed. Examination with referral or declined status was excluded.	Used recommendations from DPCP and CDEMS technical support staff.
Annual nephropathy screening (have at least 1 lab result for any of the following tests in 2004: urinary albumin/microalbumin, serum albumin/microalbumin, 24-hour urine protein, or microalbumin-to-creatinine ratio)	Urinary albumin/microalbumin: 0.1-600 mg Serum albumin/microalbumin: 1-6 g/dL Urinary creatinine: 1-30,000 mg/dL Serum creatinine: 0.1-20 mg/dL Albumin/creatinine ratio: 0.03-600,000 µg/mg or 0.00003-600 µg/mg^b^ 24-hour urine protein: 3-2,000 mg/dL	Excluded patients with nephropathy diagnosis before 2004 or before nephropathy test in 2004. Because there is no standard way of reporting nephropathy results in CDEMS registries, nonnumeric results were subject to manual review by project epidemiologists.^c^	Contacted Quest Diagnostics national reference lab for valid reporting ranges.
Annual LDL cholesterol test and control (have at least 1 lab result for LDL or non-HDL cholesterol in 2004)	LDL cholesterol: 10-350 mg/dL HDL cholesterol: 5-290 mg/dL Total cholesterol: 40-1,000 mg/dL Non-HDL cholesterol: 35-710 mg/dL^d^	Not applicable	Contacted Quest Diagnostics national reference lab for valid reporting ranges (confirmed ranges with Washington State DOH and public health laboratories).
Annual blood pressure screen and control (have at least 1 measurement result for blood pressure in 2004)	Systolic: 60-300 mm Hg (lower limit recommended by Washington State DOH consultants) Diastolic: 0-280 mm Hg (upper limit calculated from limit on difference between systolic and diastolic blood pressure values)	Systolic blood pressure had to be greater than diastolic blood pressure. Both systolic and diastolic values were not null. Difference between systolic and diastolic blood pressure could not be <20 mm Hg or >100 mm Hg. Type of visit coded as office visit.	Used information in NHANES 2003-2004 physicians' examination procedures manuals and recommendations from Washington State DOH consultants for valid reporting ranges. Used additional edits recommended by NHANES, DPCP, and CDEMS technical support staff when applicable to this data source.
Annual/biannual HbA1c testing and control (have at least 1 lab result for HbA1c test in 2004)	2% to 20%	A1c tests had to be at least 91 days apart to be considered separate tests.	Used information in NHANES 2003-2004 laboratory procedures manuals for valid reporting ranges. Referred to Bureau of Primary Care Health Disparities Collaborative guidelines for determining frequency of tests.

Abbreviations: ICD-9-CM, *International Classification of Diseases, 9th Revision, Clinical Modification*; DPCP, Diabetes Prevention and Control Program; LDL, low-density lipoprotein; HDL, high-density lipoprotein; DOH, Department of Health; NHANES, National Health and Nutrition Examination Survey; HbA1c, hemoglobin A1c.

a All services and results had to have a corresponding date that was between January 1, 2004, and December 31, 2004.

b Calculated by dividing urinary albumin range by urinary creatinine range; urinary albumin multiplied by 1,000 to calculate micrograms.

c Included results listed as less than or greater than, positive, negative, within limit, zero (we assumed this meant result was negative), ratios that were listed in valid reporting ranges, and 1+ or 3+ for 24-hour urine protein.

d Calculated by subtracting HDL from total cholesterol.

**Table 2:** Comparison of Demographic Characteristics Between Adult Patients With Diabetes in the Consolidated Chronic Disease Electronic Management System (CDEMS) and Adults in the General Washington State Diabetes Population, 2004

Demographics	CDEMS Diabetes Population^a^		Washington State Diabetes Population^b^	

	Patients, No.	% (95% CI)	BRFSS Respondents, No.	% (95% CI)^c^
**Age, y**				
18-24	128	0.7 (0.6-0.9)	30	1.5 (0.9-2.4)
25-34	604	3.5 (3.2-3.8)	141	4.6 (3.8-5.6)
35-44	1,820	10.5 (10.0-10.9)	404	10.8 (9.5-12.1)
45-54	3,746	21.6 (21.0-22.2)	855	20.8 (19.3-22.5)
55-64	4,730	27.3 (26.6-27.9)	1,346	26.0 (24.4-27.6)
65-74	3,614	20.8 (20.2-21.4)	1,131	18.0 (16.7-19.3)
≥75	2,702	15.6 (15.0-16.1)	965	18.3 (17.0-19.7)
**Sex**				
Male	8,048	46.9 (46.1-47.6)	1,966	50.3 (48.4-52.2)
Female	9,117	53.1 (52.4-53.9)	2,909	49.7 (47.8-51.6)
**Race/ethnicity**				
White	6,281	36.2 (35.5-36.9)	4,231	83.5 (81.9-85.0)
African American	830	4.8 (4.5-5.1)	120	3.5 (2.8-4.5)
Asian	668	3.9 (3.6-4.1)	76	2.3 (1.7-3.0)
Native Hawaiian or Other Pacific Islander	231	1.3 (1.2-1.5)	30	1.1 (0.6-1.7)
American Indian or Alaska Native	273	1.6 (1.4-1.8)	112	2.5 (1.9-3.2)
Hispanic	2,276	13.1 (12.6-13.6)	235	5.3 (4.5-6.3)
Other race	29	0.2 (0.1-0.2)	14	0.4 (0.2-0.7)
Unknown	6,761	39.0 (38.2-39.7)	57	1.4 (1.0-2.0)
**Health care coverage**				
Yes	11,883	68.5 (67.8-69.2)	4,509	91.1 (89.9-92.2)
No	2,118	12.2 (11.7-12.7)	360	8.8 (7.7-10.0)
Unknown	3,348	19.3 (18.7-19.9)	<10^d^	—

Abbreviations: CI, confidence interval; BRFSS, Behavioral Risk Factor Surveillance System.

a CDEMS, 2004, Washington State Department of Health. Missing or out-of-range values were excluded.

b Washington State BRFSS, 2003-2005, Washington State Department of Health. Unknown, refused, and missing responses were excluded.

c Calculated using the binomial Wald method.

d Number of responses was not sufficient to calculate reliable estimates.

**Table 3:** Distribution of Diabetes Care Among Adult Patients With Diabetes in Chronic Disease Electronic Management System (CDEMS) Registries, Washington State, 2004

**Diabetes Care Process**	**Overall**		**Across Clinics**	

	n/N	% (95% CI)^a^	Median, %	Range, %
At least 1 HbA1c test in past year	15578/17349	89.8 (89.3-90.2)	90.4	61.5-100.0
At least 2 HbA1c tests in past year	9352/17349	53.9 (53.2-54.6)	49.6	28.3-86.1
Foot examination in past year	9165/17349	52.8 (52.1-53.6)	54.5	6.0-84.4
Eye examination in past year	6143/17349	35.4 (34.7-36.1)	28.4	3.0-59.1
Nephropathy screening in past year^b^	7184/15628	46.0 (45.2-46.8)	42.7	3.1-83.3
LDL test in past year^c^	12843/17349	74.0 (73.4-74.7)	76.3	51.6-98.1
Blood pressure screening in past year	14787/17349	85.2 (84.7-85.8)	93.3	44.2-99.0

Abbreviations: CI, confidence interval; HbA1c, hemoglobin A1c; LDL, low-density lipoprotein cholesterol.

a Calculated using the binomial Wald method.

b Missing or out-of-range values were excluded, resulting in a different denominator.

c Includes patients with non-high density lipoprotein (HDL) cholesterol test (calculated by subtracting HDL from total cholesterol) in past year.

**Table 4:** Distribution of Intermediate Health Outcomes Among Adult Patients With Diabetes in Chronic Disease Electronic Management System (CDEMS) Registries, Washington State, 2004

**Intermediate Health Outcome**	Overall		Across Clinics	

	n/N^a^	% (95% CI)^b^	Median, %	Range, %
Last HbA1c test <7.0%	8,045/15,578	51.6 (50.9-52.4)	52.0	33.3-79.2
Last HbA1c test <8.0%	11,606/15,578	74.5 (73.8-75.2)	75.9	57.7-91.7
Last blood pressure reading <130/80 mm Hg	5,030/14,787	34.0 (33.3-34.8)	32.5	13.1-53.2
Last blood pressure reading <140/90 mm Hg	9,958/14,787	67.3 (66.6-68.1)	64.8	32.4-79.4
Last LDL cholesterol test <100 mg/dL^c^	7,253/12,843	56.5 (55.6-57.3)	55.7	33.3-74.5
Last LDL cholesterol test <130 mg/dL^d^	10,509/12,843	81.8 (81.2-82.5)	81.4	66.7-91.6

Abbreviations: CI, confidence interval; LDL, low-density lipoprotein; HbA1c, hemoglobin A1c.

a Different denominators in this column reflect missing or out-of-range values that were not used in calculations.

b Calculated using the binomial Wald method.

c Includes patients with last non-high density lipoprotein (HDL) cholesterol test (calculated by subtracting HDL from total cholesterol) <130 mg/dL.

d Includes patients with last non-HDL cholesterol test <160 mg/dL.

**Table 5:** Values for Intermediate Health Outcomes Among Adult Patients With Diabetes in Chronic Disease Electronic Management System (CDEMS) Registries, Washington State, 2004

**Intermediate Health Outcome**	**Median**	**Mean (SD)**	**Range**
Last HbA1c test (%)	6.9	7.3 (1.7)	2-19
Last systolic blood pressure reading (mm Hg)	130.0	130.0 (16.7)	70-210
Last diastolic blood pressure reading (mm Hg)	78.0	76.5 (10.7)	36-140
Last LDL test (mg/dL)	98.0	101.8 (33.5)	10-297
Last non-HDL test (mg/dL)^a^	132.0	138.2 (42.1)	38-683

Abbreviations: SD, standard deviation; HbA1c, hemoglobin A1c; LDL, low-density lipoprotein cholesterol; HDL, high-density lipoprotein cholesterol.

a "Non-HDL" is calculated by subtracting HDL from total cholesterol.

**Table 6:** Comparison of Diabetes Outcomes From the Consolidated Chronic Disease Electronic Management System (CDEMS)^a^ With Outcomes From Other Data Sources

	CDEMS, %	**Comparison Data, %**	**Source**
**Diabetes Care Process**			
Foot examination in past year	52.8	74.1	WA BRFSS^b^
Eye examination in past year	35.4	70.2	WA BRFSS
		48.8	Commercial HEDIS^c^
		44.1	Medicaid HEDIS^c^
		64.2	Medicare HEDIS^c^
		58.0	NHIS^d^
At least 1 HbA1c test in past year	89.8	91.8	WA BRFSS
		84.6	Commercial HEDIS
		73.9	Medicaid HEDIS
		87.3	Medicare HEDIS
At least 2 HbA1c tests in past year	53.9	75.2	WA BRFSS
LDL cholesterol test in past year^e^	74.0	88.4	Commercial HEDIS
		74.8	Medicaid HEDIS
		90.6	Medicare HEDIS
Nephropathy screening in past year	46.0	48.3	Commercial HEDIS
		43.1	Medicaid HEDIS
		52.6	Medicare HEDIS
**Intermediate Health Outcome**			
Last HbA1c test <7.0%	51.6	37.0	NHANES^e^
Last HbA1c test <8.0%	74.5	Data unavailable	
Last HbA1c test >9.0%	13.2	31.9	Commercial HEDIS
		49.5	Medicaid HEDIS
		24.3	Medicare HEDIS
Last blood pressure reading <130/80 mm Hg	34.0	35.8	NHANES
Last blood pressure reading <140/90 mm Hg	67.3	Data unavailable	
Last LDL test <100 mg/dL^f^	56.5	34.8	Commercial HEDIS
		27.3	Medicaid HEDIS
		41.2	Medicare HEDIS
Last LDL test <130 mg/dL^g^	81.8	60.5	Commercial HEDIS
		47.0	Medicaid HEDIS
		66.9	Medicare HEDIS

Abbreviations: LDL, low-density lipoprotein cholesterol; HbA1c, hemoglobin A1c.

a Washington State Department of Health, 2004.

b Washington State Behavioral Risk Factor Surveillance System, 2003-2005, Washington State Department of Health.

c Healthcare Effectiveness Data and Information Set 2004, National Committee for Quality Assurance.

d National Health Interview Survey (NHIS), 2003, National Center for Health Statistics.

e National Health and Nutrition Examination Survey, 1999-2000, National Center for Health Statistics.

f Includes patients with non-high density lipoprotein cholesterol (HDL) test (calculated by subtracting HDL from total cholesterol) in past year.

g Includes patients with last non-HDL cholesterol test <160 mg/dL.

## Appendix. Project Costs, Time, and Tasks for Consolidating Chronic Disease Electronic Management System (CDEMS) Registry Data to Describe Diabetes Care in Washington State

### Epidemiology staff

Estimated time (hours): 1680

Estimated cost: $46,800

Tasks:

1. Designed and documented project.
2. Reviewed possible data sources.
3. Learned registry program and technical functions.
4. Defined objectives to be measured for the purposes of database manipulation.
5. Coordinated compliance with Health Insurance Portability and Accountability Act regulations.
6. Coordinated information technology needs and confidentiality of patients.
7. Coordinated human subjects and institutional review board considerations.
8. Organized logistics of CDEMS clinic recruitment.
9. Reviewed inclusion and exclusion criteria for identification of study population.
10. Coordinated CDEMS Technical Support and Consolidation Technical Support contractors.
11. Conducted preliminary CDEMS analysis and programming before receipt of final aggregated dataset.
12. Worked with contractors to determine acceptable value ranges for data analysis.
13. Set up final aggregate CDEMS dataset.
14. Cleansed and sorted dataset after contractors submitted the final dataset.
15. Analyzed and documented data.
16. Prepared and disseminated results.

### Diabetes Prevention and Control Program staff

Estimated time (hours): 360

Estimated cost: $8,400

Tasks:

1. Defined objectives to be measured for the purposes of database manipulation.
2. Wrote statement of work for contractors.
3. Analyzed and administered budget.
4. Organized logistics of CDEMS clinic recruitment.
5. Coordinated letters sent to CDEMS clinics.
6. Received and transferred databases.
7. Cleansed data and determined acceptable value ranges for data analysis.
8. Coordinated resources and personnel involved in dissemination of results.
9. Prepared and disseminated results.

### CDEMS technical support and consultant contractors

Estimated time (hours): not applicable — reimbursed for products

Estimated cost: $37,000

Tasks:

1. Identified key indicators that must be captured and available for analysis.
2. Created a standard set of instructions for clinics that describes how to assemble and transfer the CDEMS database.
3. Assisted clinics with the CDEMS database transfer.
4. Maintained confidentiality of patients during data transfer, cleansing, and aggregation.
5. Sent out e-mail reminders to clinics to request data if there was no response to the first recruitment letter.
6. Received CDEMS databases and created identification of the source clinic.
7. Worked with the technical support contractor to convert any Diabetes Electronic Management System (DEMS) databases (an earlier version of CDEMS ) to the current version of CDEMS.
8. Created a standard set of instructions for data transfer of CDEMS clinic data.
9. Created a list of clinics that sent their database.
10. Documented changes made to original datasets.
11. Submitted original individual clinic databases for analysis by epidemiologists.
12. Submitted final cleansed aggregated CDEMS dataset for analysis by epidemiologists.
